# The Mycotoxin Deoxynivalenol (DON) Promotes *Campylobacter jejuni* Multiplication in the Intestine of Broiler Chickens With Consequences on Bacterial Translocation and Gut Integrity

**DOI:** 10.3389/fvets.2020.573894

**Published:** 2020-12-09

**Authors:** Daniel Ruhnau, Claudia Hess, Bertrand Grenier, Barbara Doupovec, Dian Schatzmayr, Michael Hess, Wageha A. Awad

**Affiliations:** ^1^Clinic for Poultry and Fish Medicine, Department for Farm Animals and Veterinary Public Health, University of Veterinary Medicine, Vienna, Austria; ^2^BIOMIN Research Center, Tulln, Austria

**Keywords:** deoxynivalenol, *Campylobacter jejuni*, translocation, Ussing chamber, intestinal permeability, broiler chickens

## Abstract

Deoxynivalenol (DON) is one of the major health concern in poultry production as it targets epithelial cells of the gastrointestinal tract and contributes to the loss of the epithelial barrier function. It is well-documented that DON severely compromises various important intestinal functions in coincidence with aggravated clinical symptoms in livestock. In addition, a prolonged persistence of intestinal pathogens (e.g., *Salmonella, Clostridium*) in the gut has also been reported in pigs and chickens, respectively. Similar to DON, recent studies demonstrated that an experimental *Campylobacter* infection has severe consequences on gut health. Through experimental infection, it was found that *Campylobacter (C.) jejuni* negatively affects the integrity of the intestine and promotes the translocation of bacteria from the gut to inner organs. So far, no data are available investigating the simultaneous exposure of DON and *C. jejuni* in broilers albeit both are widely distributed. Thus, the aim of the present study was to explore the interaction between DON and *C. jejuni* which is of a significant public and animal health concern as it may affect the prevalence and the ability to control this pathogen. Following oral infection of birds at 14 days of age with *C. jejuni* NCTC 12744, we show that the co-exposure to DON and *C. jejuni* has a considerable consequence on *C. jejuni* loads in chicken gut as well as on gut permeability of the birds. A reduced growth performance was found for DON and/or *C. jejuni* exposed birds. Furthermore, it was found that the co-exposure of DON and *C. jejuni* aggravated the negative effect on paracellular permeability of the intestine already noticed for the bacteria or the mycotoxin alone by the Ussing chamber technique at certain times or intestinal segments. Furthermore, the increased paracellular permeability promotes the translocation of *C. jejuni* and *E. coli* to inner organs, namely liver and spleen. Interestingly, *C. jejuni* loads in the intestine were higher in DON-fed groups indicating a supportive growth effect of the mycotoxin. The actual study demonstrates that co-exposure of broiler chickens to DON and *C. jejuni* has not only considerable consequences on gut integrity but also on bacterial balance. These findings indicate that the co-exposure of broiler chickens to DON and *C. jejuni* could have a significant impact on gut health and bacteria translocation leading to an increased risk for public health.

## Introduction

The global contamination of food and feed with mycotoxins is a crucial problem, and the presence of mycotoxins in poultry feeds represents a constant threat to the poultry industry and production losses. Deoxynivalenol (DON) is the most widespread trichothecene mycotoxin in feeds. The main mode of action of DON is the inhibition of protein synthesis, which primarily affects rapidly dividing cells, such as those of the gut and the immune system (1, 2). As a consequence, it was shown that DON increases the susceptibility to diseases (3, 4).

Likewise, *Campylobacter* (*C*.) *jejuni* is the most frequent cause of foodborne disease in humans. These bacteria are a major concern for the poultry industry as the prevalence of infected broiler flocks is reported with an average of 71.2% in EU countries (5). Recent studies also elucidated that *Campylobacter* can have a negative impact on broiler gut health (6–11). Mainly, it affects the integrity of the gut epithelium which results in reduction of villi height, decrease of crypt depth with negative consequences on nutrient transport and absorption. Furthermore, *C. jejuni* can spread to internal organs, because of increased intestinal permeability (10, 12, 13). In this context, it was also shown that *C. jejuni* facilitates not only the translocation of *C. jejuni* itself but also the spread of *Escherichia* (*E*.) *coli* to internal organs (14–17).

So far, only few studies have investigated the interaction between DON and enteric pathogens. It was found that the co-exposure of pigs to DON and *Salmonella* Typhimurium potentiated the inflammatory gut response, promoted *Salmonella* invasion and its translocation across the intestinal epithelium (3). Furthermore, it was shown *in vitro* that DON enhanced the translocation of a pathogenic *E. coli* strain over the intestinal epithelial cell monolayer (18). Recently, it was also demonstrated that feeding of DON is a predisposing factor for the development of necrotic enteritis in broiler chickens due to the negative influence of the mycotoxin on the epithelial barrier (4).

Consequently, the objective of the present study was to demonstrate the interaction between DON and *C. jejuni* in broiler chickens. For this purpose, the epithelial paracellular permeability on intestinal parts was determined by applying the Ussing chamber technique. In this context, Ussing chambers, an *ex vivo* technique, are increasingly being used to measure epithelial ion transport, epithelial barrier function and electrophysiological properties of living tissues (19–22). Furthermore, the colonization of *C. jejuni* in the gut as well as the translocation of *C. jejuni* and *E. coli* to inner organs were analyzed.

## Materials and Methods

### Study Design, Sampling, and Performance Data

One hundred and twenty 1-day–old male and female broiler chicks were obtained from a commercial hatchery (Ross-308; Gefluegelhof Schulz, Lassnitzhoehe, Austria) and randomly allocated to four treatment groups (30 birds/ group, divided into 5 replicates (6 birds/replicate): group 1 (control); group 2 (DON 5 mg/kg); group 3 (DON 5 mg/kg + *C. jejuni*); and group 4 (*C. jejuni*). Upon arrival, birds were weighed and tagged (Swiftack, Heartland Animal Health Inc., Fair Play, MO). The broilers were kept in floor pens on wood shavings and light was set at a 12h:12h light/dark cycle. Temperature was maintained at 35°C during the first days of life and reduced to 25°C with the age of birds. Feed and water were provided *ad libitum*. The experiment was carried out for 5 consecutive weeks.

Birds in groups 1 and 4 were fed with control diet without DON (non-contaminated diet during the starter and grower periods). Birds in groups 2 and 3 received the same diet contaminated with DON (DON 5 mg/kg). Diet was supplied from the first day without adaption period, as there is no evidence of gender differences or diet adaption period affected results in the response of gut physiology to diet as previously described (23). The control diet was prepared with non-contaminated wheat. The mycotoxin contaminated diet was prepared by replacing “non-contaminated” control wheat with DON contaminated wheat. Moreover, the 5 mg DON/kg feed in this study is the currently applicable EU guidance value of DON contamination (5 mg DON/kg poultry feed) (24, 25). Consequently, the used feeding model is relevant to the field situation. The composition of the diet comprised maize, wheat, soy, soybean meal, soybean oil, and rapeseed oil. Additionally, a premix of vitamins, minerals, mono calcium phosphate, and salt was supplemented. Feeds were provided by Biomin Holding GmbH (Tulln, Austria). Starter diet was fed for 9 days, followed by the grower diet from day 10 until 35 days of age.

Cloacal swabs were taken on a weekly basis starting from 1st day of life until the end of the trial. At 7, 14, and 21 dpi, cloacal swabs were taken to monitor *C. jejuni* excretion and shedding. These swabs were streaked directly onto modified charcoal-cefoperazone-deoxycholate agar (CM0739, OXOID, Hampshire, UK) to determine the *Campylobacter* status. Plates were incubated under microaerophilic conditions at 41.5°C for 48 h (Genbox microaer, BioMerièux, Vienna, Austria).

*C. jejuni* reference strain NCTC 12744 was cultured in LB medium (Lennox L broth base, Thermo Fisher Scientific Inc., Invitrogen by Life Technologies Corporation, Waltham, MA) at 41.5°C for 48 h under microaerobic conditions using GENbox microaer bags (BioMerieux, Vienna, Austria). Thereafter, the bacteria were enumerated by preparing 10-fold dilutions in phosphate-buffered saline with a PH of 7.4 (PBS, Thermo Fisher Scientific Inc., Gibco Life Technologies Corporation) and plated on Campylosel agar (BioMerieux, Vienna, Austria), followed by microaerobic incubation at 41.5°C for 48 h.

At day 14, birds of groups 3 and 4 were orally inoculated via feeding tube (gavage) with 1-ml of a PBS suspension containing 1 × 10^8^ CFU/ml of *C. jejuni* reference strain NCTC 12744. The group 1 (DON and pathogen free group) and group 2 (DON-only) received a sham infection with 1 ml of a PBS via feeding tube (gavage).

At 7, 14, and 21 days post infection five birds from each group (one bird/ replicate) were randomly selected and euthanized by intravenous application of Thiopental (20 mg/kg) into the wing vein followed by exsanguination via cutting of the jugular vein. Gross pathological examination was performed according to a standardized protocol (10). During post-mortem examination, samples in the following order: liver, spleen, duodenum, jejunum and caeca were aseptically collected and processed for bacteriological investigations. Furthermore, for Ussing chamber, intestinal segments were taken immediately after slaughter from the duodenum, mid-jejunum and cecum. The tissues were rinsed with ice-cooled PBS perfused with 95% O_2_ and 5% CO_2_. The underlying serosa was stripped off and the epithelial layers were mounted in Ussing chambers. The experimental procedures were performed as described below.

The following performance parameters were determined: (a) weekly individual body weight (BW) per bird; (b) weekly individual body weight gain (BWG) per bird calculated as the difference in body weight between the start and the end of each weighing period; (c) feed intake measured daily over the course of the feeding period at replicate level (5 replicates/group); (d) feed conversion ratio (FCR) calculated as relation of feed intake and body weight gain (5 replicates/group) at replicate level. The daily feed intake was calculated as the difference between the amount of feed supplied to the birds and the amount of feed left over in the morning. Until 21 days of age, the performance parameters of 30 birds/group were determined. Thereafter, 25 and 20 birds/group were used for measuring the performance parameters at 28 and 35 days of age, respectively.

### Determination of Paracellular Permeability Applying Ussing Chambers

From five birds per group at each killing time point, segments of duodenum, jejunum and cecum (2 replicates/ segment/ bird) were harvested and prepared for Ussing chamber studies to measure the paracellular mannitol fluxes. Epithelial layers had an exposed serosal area of 1.1 cm^2^ and were incubated with 12 mL of buffer solution on their mucosal and serosal sides under short-circuit conditions. Following tissue stabilization, ^14^C-mannitol (0.1 mCi/ml; Hartmann Analytic) was added to the mucosal solution. After a 30-min equilibration period, at the beginning and at the end of the experiment, 100 μl were taken from the “hot side” of each chamber in order to calculate the specific activity of this Ussing chamber from the mean value of the samples. Thereafter, during the course of the experiment, 0.6-mL samples were taken from the serosal compartment (cold side) at defined time intervals (every 30-min). Manipulation of Ussing chambers and experimental procedures were performed as described by Awad et al. (26). Finally, after addition of a liquid scintillation fluid to all samples up to 5 mL, the presence of radioisotope in the samples was measured by a liquid scintillation counter (Aquasafe 300 Plus, Zinsser Analytic, Maidenhead, UK).

### Bacterial Enumeration

For bacterial enumeration, 1 g of tissue samples from five birds per group (one bird/ replicate) at each killing time point was collected. The samples were homogenized (Ultra-Turrax IKA, Staufen, Germany) in 1:10 (wt:vol) phosphate-buffered saline (PBS). Afterwards serial ten-fold dilutions were prepared from the stock suspension. Serial dilutions varied for each organ as varying numbers of bacteria were expected (for liver and spleen from 10^0^ up to10^2^; duodenum from 10^0^ up to 10^4^; jejunum from 10^2^ up to 10^4^; and cecum from 10^4^ up to 10^10^).

*C. jejuni* reference strain NCTC 12744 was cultivated at 41.5°C for 48 h under microaerophilic conditions (5% O_2_, 10% CO_2_, 85% N_2_) on CASA agar (BioMerièux, Vienna, Austria) or modified charcoal-cefaprazone-deoxycholate (mCCD) agar (CM0739, OXOID, Hampshire, UK). *Escherichia coli* was aerobically grown at 37°C for 24 h on MacConkey agar (selective medium, Neogen, Heywood, UK) (16). Enumeration of *C. jejuni* (red colonies) and *E. coli*. (pink colonies) from intestinal samples was performed by plating 100 μl of each dilution on CASA agar (BioMerièux, Vienna, Austria) and MacConkey agar in duplicate, respectively. To determine the translocation of *C. jejuni* and *E. coli* in liver and spleen, 100 μl of each dilution was also plated in duplicate on either CASA or MacConkey agar, respectively. CFU counts were determined by calculating the mean value of the both plates. Generally, the suitable colony counting range was <300 CFU on plate.

### Statistical Analysis

All statistical analyses were performed using IBM SPSS statistics 24, SPSS software (Chicago, IL, USA). Data are presented as means with standard error of mean (SEM). To evaluate the normality Kolmogorov-Smirnov's test was utilized. A multivariate general linear model, ANOVA, Duncan's multiple range test and LSD were performed to analyze performance, bacterial translocation and mannitol flux data. Furthermore, statistical analysis of *in vitro* bacterial growth data for significant differences between the two groups was performed using Student's *t*-test. *P*-values of ≤ 0.05 were considered significant.

## Results

### Birds' Performance Parameters

**Figure 1:**
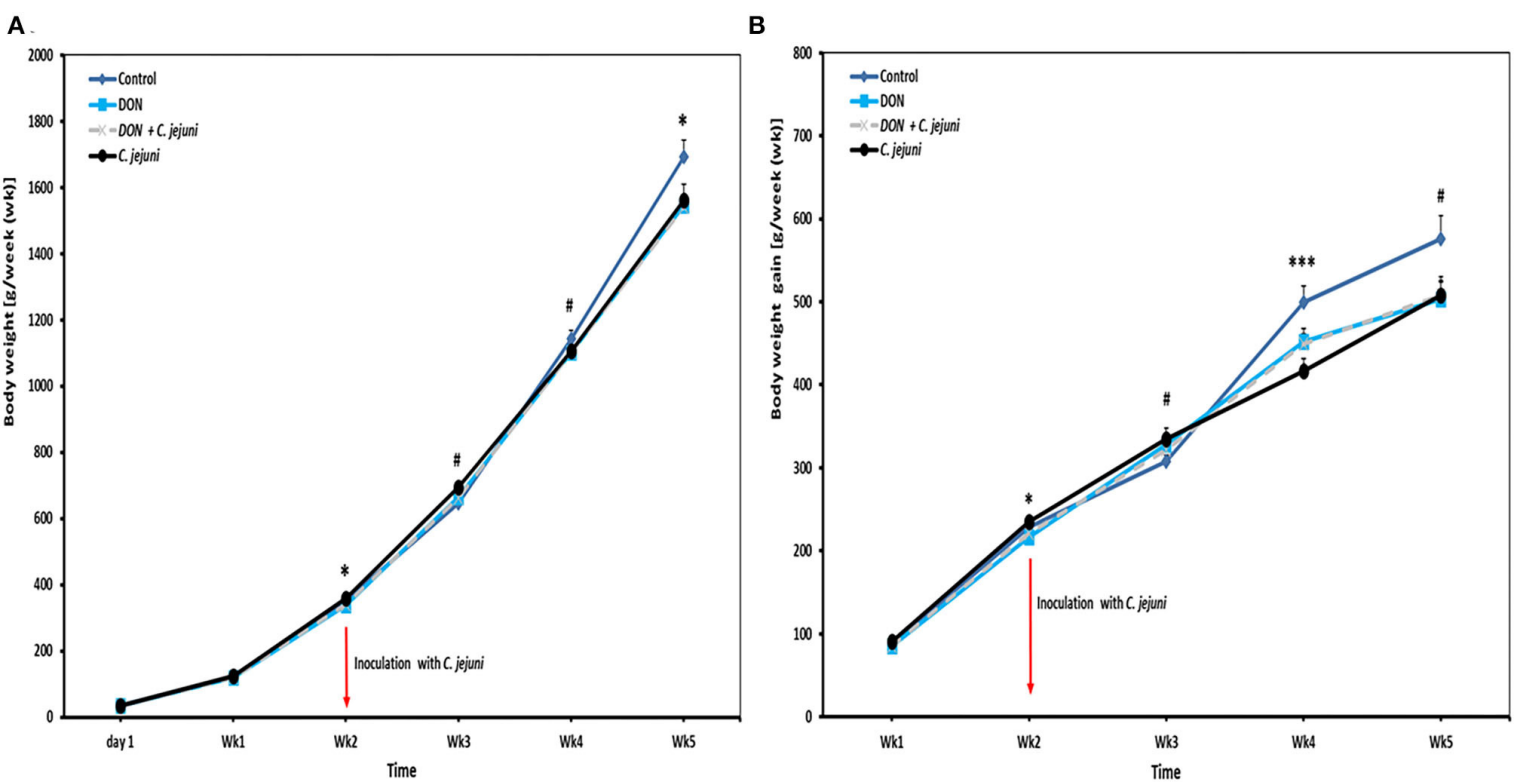
Effect of DON and/or *C. jejuni* exposure on **(A)** body weight (BW) and **(B)** body weight gain (BWG). Results are presented as mean and standard error of mean (SEM). Asterisks mark differences with ^#^*P* ≤ 0.1, *P ≤ 0.05, or ***P ≤ 0.001.

Results of the average body weights (BW, means of all individual birds) and body weight gains (BWG) of the broilers fed with either control diets or diets contaminated with DON are presented in Figures 1A,B. In general, a tendency of decreased BW was found in broilers when fed with 5 ppm DON compared to the controls (*P* < 0.1), but this effect did not reach statistical significance. However, at wk 2 and wk 5 of age, a significant decreased in BW (*P* = 0.029 and *P* = 0.050, respectively) was found in the DON-only group compared to the control group. Furthermore, at 2 and weeks 4 of age, a significantly lower BWG was seen in birds fed with DON contaminated diet with or without *C. jejuni* compared to the control group (*P* < 0.05 and *P* < 0.001). But this effect did not reach statistical significance at week 3 and 5. The overall body weight was significantly (*P* = 0.052) lower (1,509 ± 33 g) in the DON group compared with the control group (1,659 ± 48 g).

**Figure 2:**
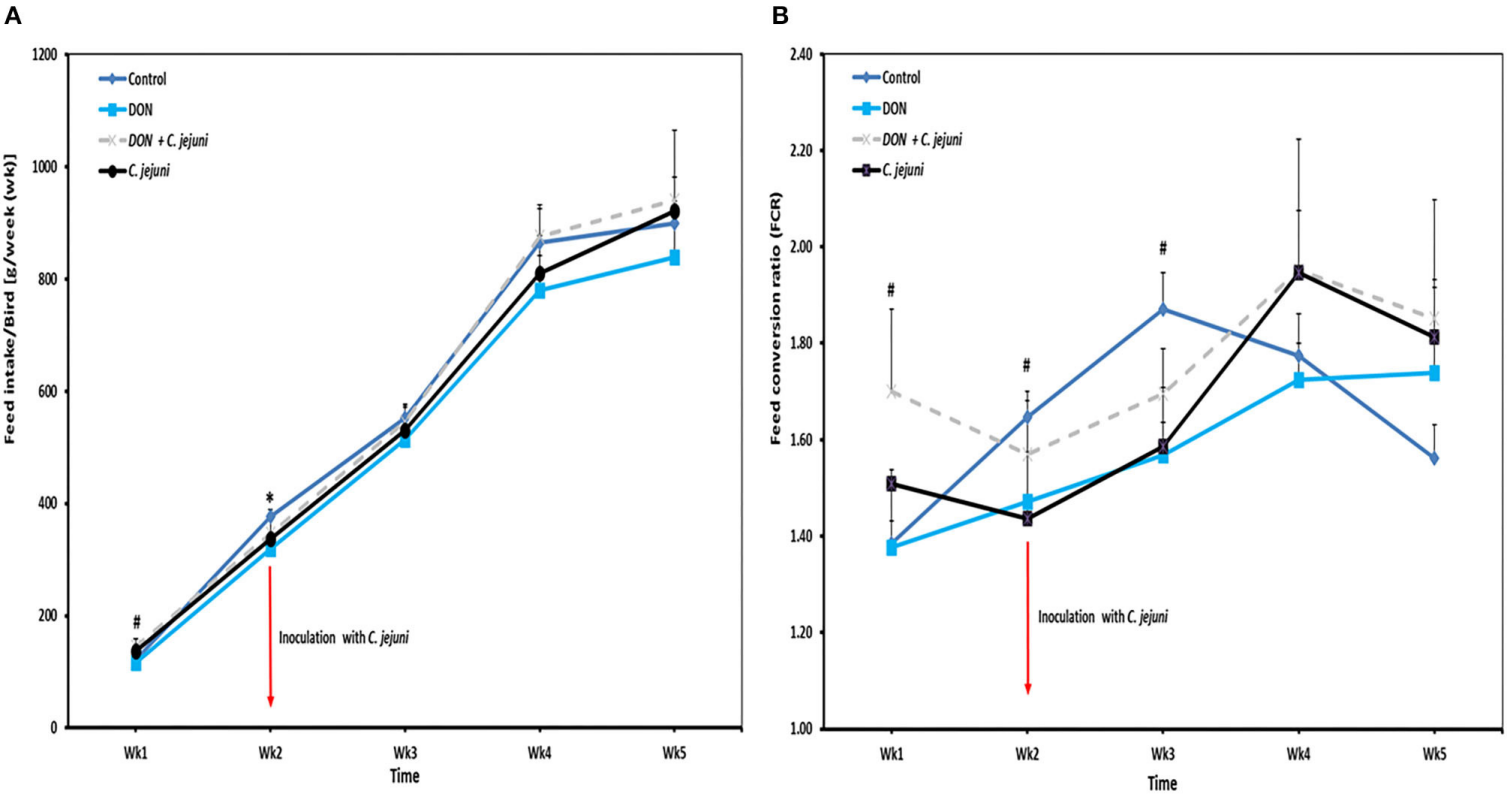
Effect of DON and/or *C. jejuni* exposure on **(A)** feed intake (FI) and **(B)** feed conversion rate (FCR). Results are presented as mean and SEM. Asterisks mark differences with ^#^*P* ≤ 0.1, or *P ≤ 0.05.

Throughout the whole experiment the feed intake was numerically lower in DON-only group compared to the other three groups (Figure 2A). In addition, slight differences were observed in the average daily feed intake between control birds (80 g/bird/d) and infected birds (73 g/bird/d). At week 2 of age, a significant decreased in feed intake (*P* = 0.041) was found in DON-only group compared to the control group. However, an increased feed intake from week 4 onwards was recorded in birds from groups 3 and 4 infected with *C. jejuni* compared to the other groups. In this context, the feed conversion rate (FCR) at wk 5 of infected birds with or without DON was higher (1.8 ± 0.10) compared with control birds (1.5 ± 0.07). Throughout the whole trial the average feed conversion ratio was lower in the group in which birds received DON contaminated diet only compared with the other three groups, but this effect did not reach statistical significance (Figure 2B).

### Intestinal Permeability

**Figure 3:**
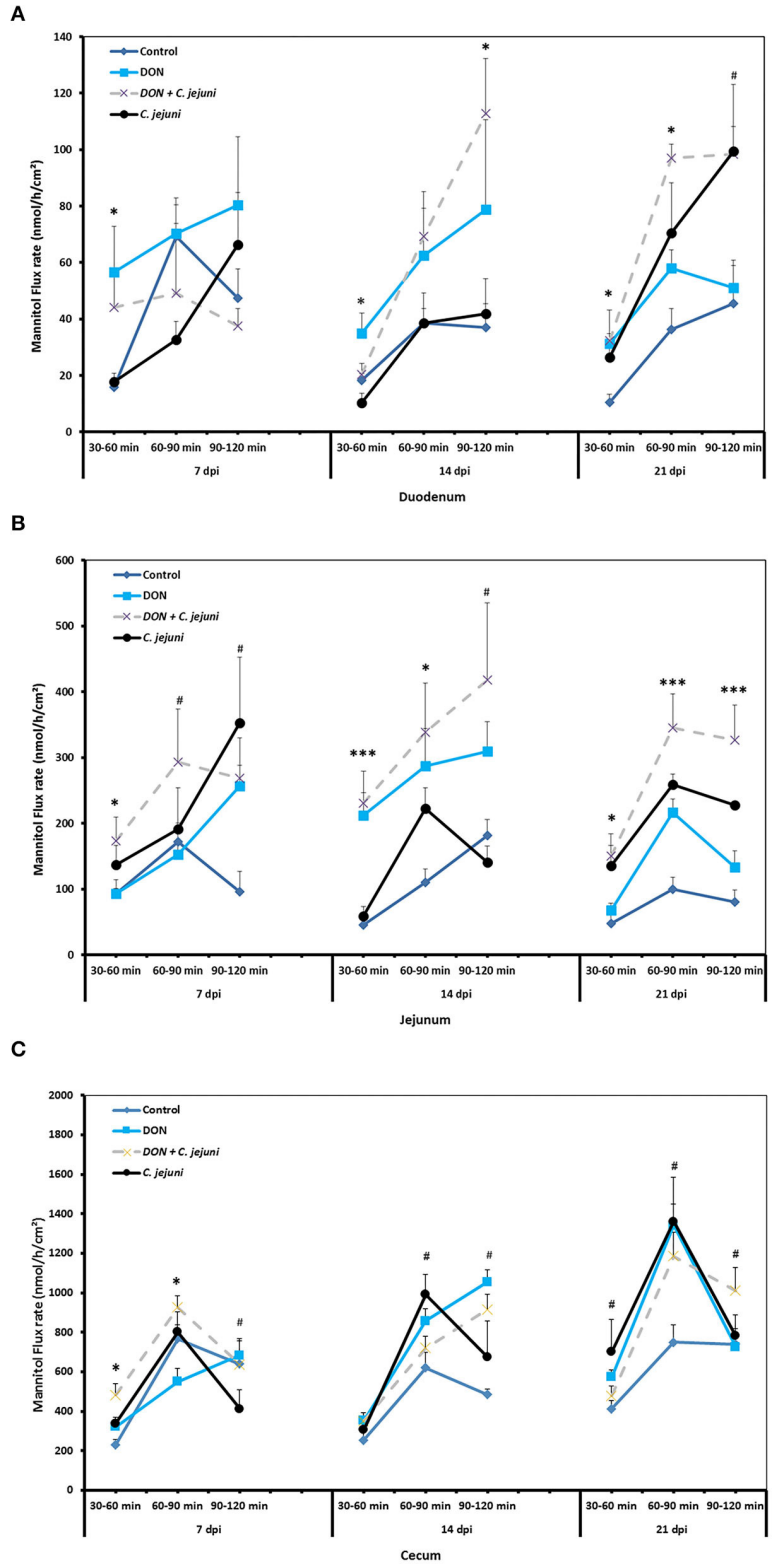
Effect of DON and/or *C. jejuni* exposure on paracellular permeability in duodenum **(A)**, jejunum **(B)**, and cecum **(C)** at different times post infection. Mucosal to serosal flux (J_ms_) of the permeability marker ^14^C-mannitol were performed in Ussing chambers. Data are presented as the mean values and SEM (*n* = 5). Asterisks mark differences with ^#^*P* ≤ 0.1, *P ≤ 0.05, or ***P ≤ 0.001.

The unidirectional flux of ^14^C-mannitol (paracellular marker) in duodenum, jejunum and cecum is shown in Figures 3A–C. During the baseline period (30–60 min), at 7, 14, and 21 dpi, there were significant differences (*P* < 0.05) in the flux of mannitol in the duodenum among the different groups. Moreover, the co-exposure to DON and *C. jejuni* potentiates this negative effect on the permeability of the duodenum in the second (60–90 min) and the third (90–120 min) flux periods, which is indicated by a higher mannitol flux at 14 dpi (*P* < 0.05) and 21 dpi (*P* < 0.01) compared to the controls. Similarly, the results revealed that DON exposure induces an increased mannitol flux in the jejunum and cecum at 14 and 21 dpi especially during the second flux period (60–90 min). This indicated that the increased luminal mannitol concentration from 10 to 20 mM resulted in an increase in mannitol flux most probably due to passive diffusion. The results provided evidence that DON could increase the paracellular intestinal permeability at certain time points or intestinal segments, which might explain the elevated *E. coli* translocation to the liver and spleen. Data showed that DON impact on intestinal permeability varies highly between different gut sites, which likely reflects different mechanisms for the alterations of intestinal permeability of each intestinal segment. The results also revealed that *C. jejuni* as such induces an increase in the flux of ^14^C mannitol in the jejunum and cecum at 14 and 21 dpi, and in the duodenum at 21 dpi.

### Influence of DON on Colonization and Translocation of *E. coli* and *C. jejuni*

**Figure 4:**
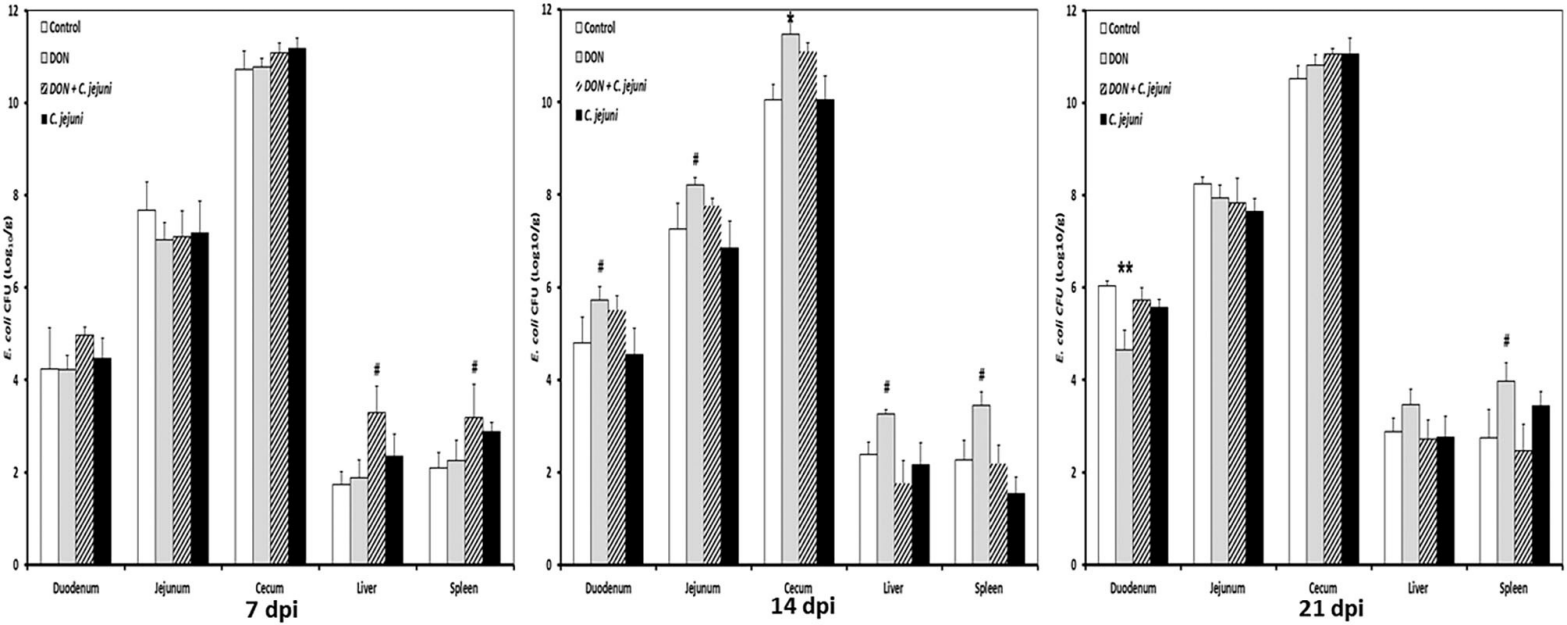
*E. coli* counts at different time points post infection from duodenum, jejunum, cecum, liver, and spleen in birds fed with either control or DON contaminated diet with or without *C. jejuni* infection. Results are presented as mean values and SEM (*n* = 5). Number of bacteria are expressed in logarithmic form of colony forming units (log CFU/g). Asterisks mark differences with ^#^*P* ≤ 0.1, *P ≤ 0.05, or **P ≤ 0.01.

Higher numbers of *E. coli* were detected in the duodenum, jejunum and cecum at 14 dpi in both DON-fed groups either with or without *C. jejuni* compared to the control group (Figure 4). A significant increase in bacterial counts was only found in cecum, while the counts for the other locations were higher as well but the statistical significance was not reached. Interestingly, at 7 dpi the translocation (translocation in all birds present) of *E. coli* to the liver and spleen was higher in group 3 (DON diet + *C. jejuni*) coinciding with the highest number of *C. jejuni* in the duodenum. However, at 14 and 21 dpi this effect could only be attributed to the presence of DON.

**Figure 5:**
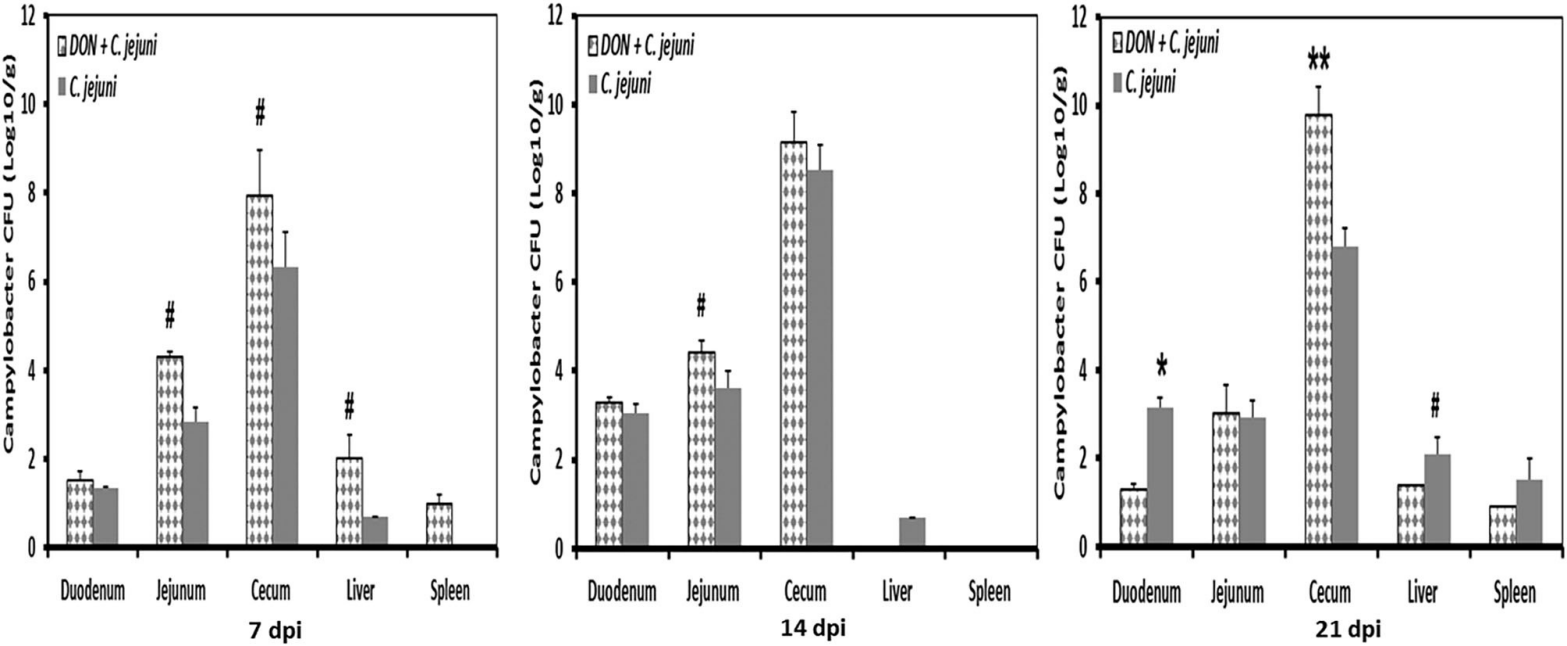
*C. jejuni* counts at different time points post infection from duodenum, jejunum, cecum, liver, and spleen of infected birds fed with either control or DON contaminated diet. Results are presented as mean values and SEM (*n* = 5). Numbers of bacteria are expressed in logarithmic form of colony forming units (log CFU/g). Asterisks mark differences with ^#^*P* ≤ 0.1, or *P ≤ 0.05.

No *Campylobacter jejuni* were detected in swab samples taken from day-old birds and prior to infection at 14 days of age. Non-infected birds stayed *Campylobacter jejuni*-negative throughout the experiment. In infected groups, shedding of *Campylobacter* was detected in almost all infected birds (90%). Fecal droppings remained normal in both control and infected birds, with no signs of diarrhea over the course of the animal trial. A tendency of increased *Campylobacter* load in the jejunum at 7 dpi and 14 dpi as well as in cecum at 7 dpi was recognized in birds fed with DON contaminated diet (Figure 5). This increase was more pronounced in the ceca 21 dpi (*P* < 0.01). Furthermore, the presence of DON in the diet led to an increase in *Campylobacter* translocation to liver and spleen at 7 dpi, but this effect did not reach statistical significance. The results demonstrated that the translocation of *C. jejuni* to liver and spleen varied depending on sampling time point postinfection, as indicated by the detection of *C. jejuni* in 3/5 infected birds at 7 and 21 dpi but not at 14 dpi.

## Discussion

Both, mycotoxin contamination of feed and *C. jejuni* prevalence in broilers have an increasing global health and economic impact on poultry production. The *Fusarium* mycotoxin DON is a ubiquitous mycotoxin with negative effects on the growth performance of broiler chickens (26, 27). As an inhibitor of protein synthesis DON mainly affects cells with a high-protein turnover, such as intestinal epithelial and immune cells (28). The intestinal mucosa acts as an effective defensive barrier against the invasion of harmful substances and counteracts the occurrence of intestinal diseases (29). The negative impact of DON on the gut barrier has been associated with destruction of intestinal architecture, inhibition of activity of intestinal stem cells and changes in the modulation of tight junction protein expression (2, 18, 26, 30–35). Similarly, effects of *C. jejuni* on the gut physiology of chickens have already been reported. It was shown that these bacteria have a negative impact on the nutrient absorption indicating that a lower slaughter weight might probably be due to the reduction in feed efficiency (13). Furthermore, the occurrence of a leaky gut syndrome caused by *C. jejuni* is known by enhancing bacterial translocation from the gut to internal organs (16).

Vanderbroucke et al. (3) reported that the exposure of pigs to DON and *S*. Typhimurium promoted *Salmonella* invasion in the gut and its translocation across the intestinal epithelium. Similarly, an increased translocation of *E. coli* following DON exposure was reported in human and porcine cell monolayers *in vitro* and in chickens *in vivo* (18, 26, 36). So far, no data are available on the co-exposure of DON and *C. jejuni* in broiler chickens, the purpose of the actual study.

The study indicates that the co-exposure of broiler chickens to DON and *C. jejuni* supported the *C. jejuni* colonization in the gut at certain time points post-infection, revealing that DON might provide a favorable condition for *Campylobacter* growth. Furthermore, an indirect mechanism should not be excluded as DON can induce a leakage of plasma amino acids or proteins into the intestinal lumen (4, 20). The mode of action, either direct or indirect, needs to be determined in future studies to elucidate whether such substances provide the necessary growth substrate for extensive proliferation of *Campylobacter*. Understanding how mycotoxins influence prokaryotes began to emerge as an important area of future research perspectives (37, 38). Recently presented evidence indicates that DON can negatively affect the gut microbiota of either humans or animals (39, 40). This, in turn, has led to a greater interest in understanding bacterial responses toward DON.

Independent of this, the co-exposure promotes the translocation of *C. jejuni* and *E. coli* to the liver and spleen at certain time points post infection. In this context, the results also indicated that DON increased the intestinal paracellular permeability as reported in our previous study (26). However, in the current study, in addition to jejunum and cecum, the permeability of the duodenum was impaired at different time points demonstrating a detrimental effect of DON on the barrier function of the entire intestine.

The study also revealed that the co-exposure to DON and *C. jejuni* potentiates a significant increase in paracellular permeability as both, pathogen and mycotoxin, affected barrier functions of the intestine. Thus, the presented data indicated that increased gut paracellular permeability could be associated with a higher translocation of *C. jejuni* and *E. coli* at certain time points. In context with the previous results (26), the present study showed that DON in a concentration of 5 mg/kg could have a negative effect on the growth performance of chickens. Those results are of practical relevance as the current guidance for the tolerated value of DON in poultry diets is set at 5 mg/kg feed (European Commission No. 1881/2006).

Taken altogether, results of the actual studies strengthen the hypothesis that DON could influence the infection profile of *C. jejuni*, a subject not demonstrated so far. Indeed, it was found that the co-exposure of broilers to DON and *C. jejuni* can potentiate gut permeability, promote *Campylobacter* colonization and its translocation across the intestinal epithelium. This in turn will lead to a greater interest in understanding bacterial responses toward DON, and the involved mechanisms in order to build a more comprehensive picture of DON-induced changes in prokaryotes, highlighting the need for further investigations of DON effects on prokaryotes.

## Data Availability Statement

The raw data supporting the conclusions of this article will be made available by the authors, without undue reservation.

## Ethics Statement

The animal study was reviewed and approved by the institutional ethics committee of the University of Veterinary Medicine and the Ministry of Research and Science under the license number GZ -68.205/0179-V/3b/2018.

## Author Contributions

All authors listed have made a substantial, direct and intellectual contribution to the work, and approved it for publication.

## Conflict of Interest

The authors declare that the research was conducted in the absence of any commercial or financial relationships that could be construed as a potential conflict of interest.
